# Vitamin C: A Novel Regulator of Neutrophil Extracellular Trap Formation

**DOI:** 10.3390/nu5083131

**Published:** 2013-08-09

**Authors:** Bassem M. Mohammed, Bernard J. Fisher, Donatas Kraskauskas, Daniela Farkas, Donald F. Brophy, Alpha A. Fowler, Ramesh Natarajan

**Affiliations:** 1 Department of Pharmacotherapy and Outcomes Science, Virginia Commonwealth University, Richmond, VA 23298, USA; E-Mails: mohammedbm@vcu.edu (B.M.M.); dbrophy@vcu.edu (D.F.B.); 2 Department of Clinical Pharmacy, Faculty of Pharmacy, Cairo University, Cairo 11562, Egypt; 3 Division of Pulmonary Disease and Critical Care Medicine, Virginia Commonwealth University, Richmond, VA 23298, USA; E-Mails: bjfisher@vcu.edu (B.J.F.); dkraskauskas@vcu.edu (D.K.); dfarkas3@vcu.edu (D.F.); afowler@mcvh-vcu.edu (A.A.F.)

**Keywords:** vitamin C, sepsis, neutrophils, NETosis, cell free DNA, nuclear factor κB

## Abstract

**Introduction**: Neutrophil extracellular trap (NET) formation was recently identified as a novel mechanism to kill pathogens. However, excessive NET formation in sepsis can injure host tissues. We have recently shown that parenteral vitamin C (VitC) is protective in sepsis. Whether VitC alters NETosis is unknown. **Methods**: We used Gulo^−/−^ mice as they lack the ability to synthesize VitC. Sepsis was induced by intraperitoneal infusion of a fecal stem solution (abdominal peritonitis, FIP). Some VitC deficient Gulo^−/−^ mice received an infusion of ascorbic acid (AscA, 200 mg/kg) 30 min after induction of FIP. NETosis was assessed histologically and by quantification for circulating free DNA (cf-DNA) in serum. Autophagy, histone citrullination, endoplasmic reticulum (ER) stress, NFκB activation and apoptosis were investigated in peritoneal PMNs. **Results**: Sepsis produced significant NETs in the lungs of VitC deficient Gulo^−/−^ mice and increased circulating cf-DNA. This was attenuated in the VitC sufficient Gulo^−/−^ mice and in VitC deficient Gulo^−/−^ mice infused with AscA. Polymorphonuclear neutrophils (PMNs) from VitC deficient Gulo^−/−^ mice demonstrated increased activation of ER stress, autophagy, histone citrullination, and NFκB activation, while apoptosis was inhibited. VitC also significantly attenuated PMA induced NETosis in PMNs from healthy human volunteers. **Conclusions**: Our *in vitro* and *in vivo* findings identify VitC as a novel regulator of NET formation in sepsis. This study complements the notion that VitC is protective in sepsis settings.

## 1. Introduction

Polymorphonuclear neutrophils (PMN) play key roles in the host response to pathogens by regulating innate host defenses and modulating inflammation. PMN combat pathogens by multiple mechanisms including phagocytosis, followed by exposure to reactive oxygen intermediates (short-lived and long-lived) in phagolysosomes [1], degranulation, which involves release of anti-bacterial peptides and proteases to kill pathogens [2], as well as production of cytokines and other inflammatory mediators. Aside from these traditional mechanisms, another mechanism for pathogen killing, the formation of neutrophil extracellular traps (NETs) by NETosis, a novel cell death pathway different from apoptosis and necrosis, was recently identified [3,4]. Although NETosis plays a crucial role in host defense during local infection by trapping and killing pathogens, excessive NET formation during systemic infections becomes self-defeating by promoting tissue injury and organ damage [5].

Sepsis, a leading cause of death with high mortality rates, is characterized by excessive inflammation and exuberant immune responses that lead to increased circulating PMN levels and extensive PMN sequestration in the lung. This massive influx of PMNs to the lungs often leads to acute lung injury (ALI) [6]. One postulated mechanism by which PMNs cause ALI is NETosis [7]. In sepsis, NETs are formed in response to pro-inflammatory stimuli such as lipopolysaccharide (LPS) and interleukin-8 (IL-8) [8,9] by expulsion of genomic DNA into web-like extracellular structures that display antimicrobial proteins such as histones, neutrophil elastase, and myeloperoxidase [10]. During NETosis, various signaling pathways lead to dissolution of nuclear envelope, thus allowing the mixing of nuclear chromatin with granular antimicrobial proteins from cytoplasmic granules, and then, by releasing the DNA into lattice-like structures, NETs concentrate proteases and antimicrobial proteins in the vicinity of trapped pathogens. However, in sepsis, exposure to NETs also produces organ injury. Indeed, Dwivedi *et al.* recently showed that NETosis, as determined by the circulating cell free DNA (cf-DNA) content, could predict ICU mortality in severe sepsis better than existing severity of illness or organ dysfunction scoring systems and was also better than IL-6, thrombin, and protein C [11]. While effective targeting or inhibition of NET structures has been suggested as therapy to benefit sepsis [8], identification of agents with the potential to alter NET formation remains elusive.

Vitamin C (VitC) is an essential vitamin for humans. While its role as an endogenous antioxidant is well recognized, our recent research suggests that VitC beneficially impacts multiple pathways associated with sepsis [12]. Its pleiotropic mechanisms including attenuation of the pro-inflammatory response, enhancement of epithelial barrier function, increasing alveolar fluid clearance, and prevention of coagulation abnormalities constitute a primary line of defense that is protective in sepsis syndromes [13]. Intracellular levels of VitC in various tissues differ significantly from circulating plasma levels with high cellular concentrations considered to be indicative of essential metabolic function [14]. In particular, VitC accumulates in millimolar quantities in PMNs where it regulates neutrophil apoptosis [14,15]. We recently showed that VitC attenuated neutrophiliccapillaritis and improved survival in murine sepsis models [12,13]. However, whether VitC alters NETosis in sepsis settings remains unknown. Humans lack functional l-gulono-γ-lactone oxidase (Gulo), the final enzyme in the biosynthesis of VitC [16]. In contrast, mice express functional Gulo, resulting in tissues generally maintaining high levels of VitC. In order to translate data from VitC studies in mice to humans we have examined NETosis in septic mice lacking Gulo (Gulo^−/−^). Our studies show that VitC sufficiency attenuated NETosis in septic mice. Importantly, at a cellular level, we show that VitC deficient PMN were more susceptible to undergo NETosis through increased activation of endoplasmic reticulum (ER) stress and autophagy, processes considered vital for NETosis [17]. VitC deficient PMNs displayed increased expression of peptidylargininedeiminase 4 (PAD4), the key enzyme required for hypercitrullination of histones and chromatin decondensation [18]. Moreover, our studies show that the pro-survival transcription factor nuclear factor kappa B (NFκB) was augmented in the VitC deficient PMNs while apoptosis was suppressed. The inhibitory effect of VitC on NETosis was recapitulated in phorbolmyristate acetate (PMA) activated human PMN.

## 2. Experimental Section

### 2.1. Animals

Gulo^−/−^ mice were bred in-house from an established homozygous colony as previously described [19]. Vitamin C *sufficient* mice were fed *ad libitum* with regular chow and water supplemented with vitamin C (0.330 g/L) renewed twice per week. Vitamin C *deficient* mice were generated by reducing vitamin C supplementation (0.033 g/L) for one week, followed by complete removal of dietary vitamin C for an additional two weeks. Others have shown that this reduced supplementation significantly decreases the concentration of VitC in immune cells, plasma and organs [20,21].

#### 2.1.1. Feces Induced Peritonitis

Polymicrobial sepsis (peritonitis) was induced by intraperitoneal (i.p.) introduction of fecal stem solution into the peritoneum as described previously [13,19]. Thirty minutes after fecal challenge (45 mg/mL), some mice received i.p. injection of VitC as ascorbic acid (200 mg/kg in saline). Untreated mice received i.p. saline instead of VitC. Blood was collected 16 h later by cardiac puncture, and lungs harvested. Blood was allowed to coagulate, spun to separate serum, and stored at −80 °C for batch analysis of cell-free DNA (see below).

All animal studies were performed in accordance to the Virginia Commonwealth University Animal Care and Use Committee’s approved protocols (Protocol # AM10100, approved 15 March, 2011).

#### 2.1.2. Gulo^−/−^ Mice Were Divided into Five Groups

(1) (+): vitamin C *sufficient* mice received saline alone (0.4 mL, i.p.)
(2) **FIP**(+): vitamin C *sufficient* mice received fecal stem solution (0.4 mL, i.p.) followed 30 min later by saline (0.1 mL, i.p.)
(3) (−): vitamin C *deficient* mice received saline alone (0.4 mL, i.p.)
(4) **FIP**(−): vitamin C *deficient* mice received fecal stem solution (0.4 mL, i.p.) followed 30 min later by saline (0.1 mL, i.p.)
(5) **FIP**(−) + **AscA**: vitamin C *deficient* mice received fecal stem solution (0.4 mL, i.p.) followed 30 min later by ascorbic acid (0.1 mL, i.p.)

### 2.2. Isolation of Mouse Peritoneal Neutrophils

Induction of an enriched exudate of leukocytes in the peritoneal cavity of mice was performed by i.p. injection of 1 mL of aged, sterile 3% thioglycollate solution [16]. After 16 h, mice were euthanized, and the peritoneal cavity was flushed with 5 mL sterile Hanks’ balanced salt solution containing 1% BSA (HBSS). The leukocyte pellet containing ~80% neutrophils and ~20% macrophages was washed with HBSS and resuspended in RPMI-1640 medium. Total cell counts were determined with a hemacytometer. Leukocyte viability was assessed by trypan blue exclusion (>99%). PMNs were then purified by adherence to a plastic dish as described by Tsurubuchi *et al*. [22]. Briefly, cells from peritoneal exudate were plated into a 35-mm plastic dish and incubated at 37 °C in 5% CO_2_ in air for 10 min in HBSS. The cells were washed twice with fresh HBSS to remove non-adherent cells. Although there was loss of some PMNs, which did not adhere to the dish, this procedure eliminated most of the macrophages. Cytochemical staining of adherent cells using HARLECO^®^ Hemacolor^®^ Solution (EMD Millipore, EMD Millipore) revealed that >95% of the adherent cells were PMNs.

### 2.3. Immunofluorescence and Differential Interference Contrast Imaging of Lung NETs

Formalin fixed paraffin embedded mouse lung sections (3 µm) were rehydrated and heat induced antigen retrieval performed in 0.01 M citrate buffer pH 6.0 for 20 min. Sections were blocked with 1% normal swine serum (NSS, DAKO, Carpinteria, CA, USA) and incubated with primary antibody #1, rat anti-mouse CD41 (MWReg30, ab33661, Abcam, Cambridge, MA, USA), 1:10 diluted in 1% NSS/PBS overnight at 4 °C. Sections were then incubated with goat anti-rat Alexa Fluor^®^ 488 1:50 (Abcam) in PBS for 4 h followed by incubation with primary antibody #2, rabbit anti-myeloperoxidase (ab45977, Abcam) 1:10 diluted in PBS overnight at 4 °C. Sections were then incubated with chicken anti-rabbit Alexa Fluor^®^ 647 (Invitrogen, Life Technologies, Grand Island, NY, USA) 1:50 in PBS for 4 h followed by incubation with primary antibody #3, mouse anti-histone H2A (L88A6, Cell Signaling, Danvers, MA, USA) 1:200 in PBS overnight at 4 °C, and then finally incubated with goat anti-mouse IgG1 Alexa Fluor^®^ 594 (Invitrogen) 1:50 in PBS for 4 h. Nuclear counterstain was performed with DAPI (Invitrogen) 1:500 for 5 min and sections mounted with Slow Fade Gold (Invitrogen). Negative controls were run in parallel with nonspecific IgG or specific isotype. Confocal microscopy was performed with a Leica TCS SP2 laser scanning confocal microscopy system of the VCU Department of Anatomy and Neurobiology Microscope Facility. Separate images of optical sections were acquired with filters for Alexa Fluor^®^ (AF) 488, 594, 647 and DAPI. Images were assembled with ImageJ software.

### 2.4. RNA Isolation and Real-Time Quantitative PCR (QPCR) Analysis

Isolation of total RNA and real-time QPCR analyses were performed as described previously [12]. Primers used for QPCR are listed in Table 1.

**Table 1:** Murine primers used for Quantitative PCR (QPCR).

Name	Sequence 5′ to 3′
ATF4 forward	CCTAGGTCTCTTAGATGACTATCTGGAGG
ATF4 reverse	CCAGGTCATCCATTCGAAACAGAGCATCG
BiP forward	GTGCAGCAGGACATCAAGTTCTTGCC
BiP reverse	TTCCCAAATACGCCTCAGCAGTCTCC
CHOP forward	CACCTATATCTCATCCCCAGGAAACG
CHOP reverse	TTCCTTGCTCTTCCTCCTCTTCCTCC
EDEM forward	GCCCTTTGGTGACATGACAATTGAGG
EDEM reverse	TCATTATTGCTGTCAGGAGGAACACC
XBP-1s forward	TGAGTCCGCAGCAGGTGC
XBP-1s reverse	CAACTTGTCCAGAATGCCCAAAAGG
XBP-1un forward	AAGAACACGCTTGGGAATGGACACGC
XBP-1un reverse	ACCTGCTGCAGAGGTGCACATAGTC
PAD4 forward	ACAGGTGAAAGCAGCCAGC
PAD4 reverse	AGTGATGTAGATCAGGGCTTGG
ATG3 forward	CACCACTGTCCAACATGGC
ATG3 reverse	GTTTACACCGCTTGTAGCATGG
ATG5 forward	ACAAGCAGCTCTGGATGGG
ATG5 reverse	GGAGGATATTCCATGAGTTTCCG
ATG6 forward	CACGAGCTTCAAGATCCTGG
ATG6 reverse	TCCTGAGTTAGCCTCTTCCTCC
ATG7 forward	ACGATGACGACACTGTTCTGG
ATG7 reverse	AGGTTACAGGGATCGTACACACC
ATG8 forward	ACAAAGAGTGGAAGATGTCCG
ATG8 reverse	GGAACTTGGTCTTGTCCAGG
TNFα forward	GATGAGAAGTTCCCAAATGGC
TNFα reverse	TTGGTGGTTTGCTACGACG
IL-1β forward	CTGAACTCAACTGTGAAATGCC
IL-1β reverse	CAGGTCAAAGGTTTGGAAGC
18S forward	GATAGCTCTTTCTCGATTCCG
18S reverse	AGAGTCTCGTTCGTTATCGG

### 2.5. Western Blot Analysis

Neutrophil whole-cell and nuclear extracts were isolated for Western blot analysis as described previously [13]. Nuclear extracts were isolated using the NE-PER kit (Pierce Biotechnology, Rockford, IL, USA). Proteins were resolved by SDS polyacrylamide gel electrophoresis (4%–20%) and electrophoretically transferred to polyvinylidene fluoride membranes (0.2 μm pore size). Immunodetection was performed using chemiluminescent detection with the Renaissance Western Blot Chemiluminescence Reagent Plus (Perkin Elmer Life Sciences Inc., Boston, MA, USA). Blots were stripped using the Restore™ Western Blot Stripping Buffer (Pierce Biotechnology Inc., Rockford, IL, USA) as described by the manufacturer. Purified rabbit polyclonal antibodies to LC3B (L7543, Sigma-Aldrich), cleaved caspase-3 (#9661, Cell Signaling), caspase-3 (#9662, Cell Signaling), p62/SQSTM1 (NBP1-48320, Novus Biologicals), NFκB p65 (sc-109, Santa Cruz Biotechnology), Lamin B (sc-6216, Santa Cruz Biotechnology), and actin (sc-1616, Santa Cruz Biotechnology) were used in this study. Optical densities of antibody-specific bands were determined using Quantity One acquisition and analysis software (Bio-Rad, Hercules, CA, USA).

### 2.6. Isolation of Human Neutrophils and NETs Release

Human neutrophils were isolated by density gradient centrifugation and hypotonic lysis [23]. Cells were adjusted to 2 × 10^6^/mL in RPMI-1640, seeded onto 8-well IbiTreat µ-slides (Ibidi #80826), 0.3 mL per well, and allowed to adhere for 15 min. PMNs were VitC loaded by incubating for 1 h with 0.3 mM or 3 mM buffered ascorbic acid (Mylan Institutional LLC, Rockville, IL, USA). Neutrophils were stimulated with 50 nM PMA for three hours at 37 °C. Neutrophil conditioned media were centrifuged at 400× *g* for 5 min and the supernatants used for quantification of cf-DNA [24].

### 2.7. Immunofluorescence Staining of Human NETs

PMNs were fixed with 4% paraformaldehyde, permeabilized with 0.15% Triton X-100 in PBS, and blocked with 5% normal chicken serum (Sigma) in PBS. To stain NETs, slides were incubated with a mouse monoclonal anti-myeloperoxidase antibody (1:200; Santa Cruz sc-52707) and a secondary Alexa Fluor^®^ 488-conjugated chicken anti-mouse IgG antibody (1:200; Molecular Probes A-21200). After staining of DNA with DAPI, neutrophil-derived NET formation was visualized by immunofluorescence microscopy performed on an Olympus model IX70 inverted microscope outfitted with an IX-FLA fluorescence observation system equipped with a FITC and DAPI filter cubes (Chroma Technology, Brattleboro, VT, USA) through Uplan FI objectives (20×, 60×). Images were captured by an Olympus XM10 digital camera using CellSens imaging software (Olympus America, Melville, NY, USA).

### 2.8. Quantification of Cell Free DNA

The levels of cf-DNA in human neutrophil supernatants and mouse serum were quantified using the Invitrogen Quant-iTPicoGreendsDNA assay kit according to the manufacturer’s instructions (Life Technologies, Grand Island, NY, USA). Fluorescence intensity was measured on a SpectraMax Gemini XPS microplate reader with excitation at 490 nm and emission at 525 nm, with a 515 nm emission cutoff filter (Molecular Devices, Sunnyvale, CA, USA).

### 2.9. Statistical Analysis

Statistical analysis was performed using SAS 9.3 and GraphPad Prism 6.0 (GraphPad Software, San Diego, CA, USA). Data are expressed as mean ± SE. Results were compared using Student-Newman-Keuls test or one-way ANOVA and the *post hoc* Tukey test to identify specific differences between groups. Statistical significance was confirmed at a *p* value of <0.05.

**Figure 1:**
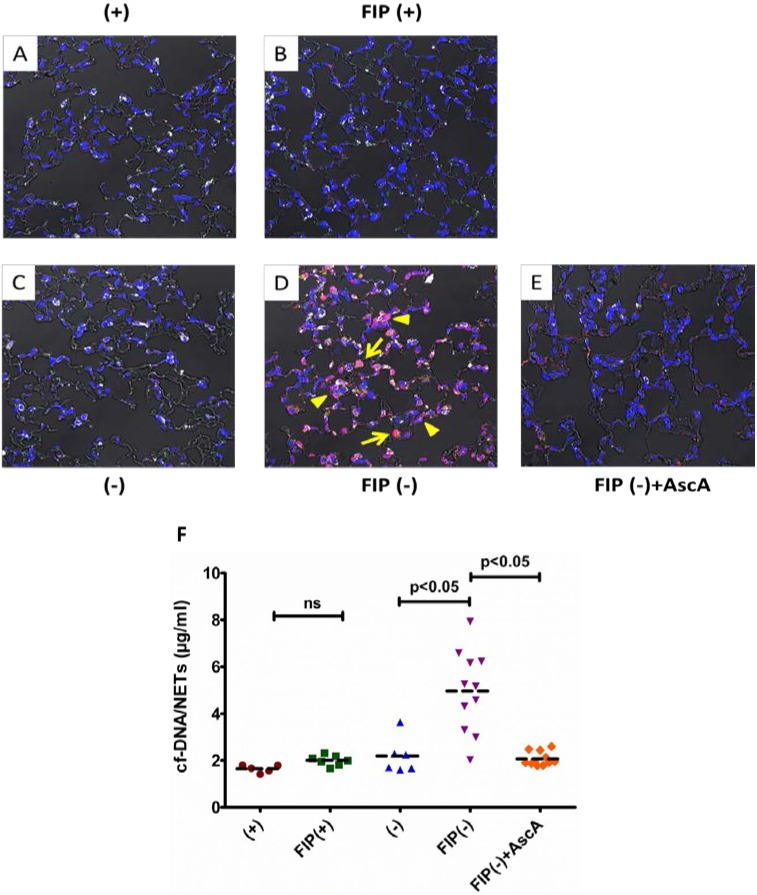
Vitamin C sufficient Gulo^−/−^ mice demonstrate reduced lung NETs and lower cf-DNA following peritonitis-induced sepsis. Representative immunofluorescence and differential interference contrast imaging of lung NETs (**A**–**E**): (**A**) VitC sufficient Gulo^−/−^ mice (+) received saline alone (0.4 mL, i.p.); (**B**) FIP exposed VitC sufficient Gulo^−/−^ mice [**FIP**(+)] received fecal stem solution (45 mg/mL, i.p.) followed 30 min later by saline (0.1 mL, i.p.); (**C**) VitC deficient Gulo^−/−^ mice (−) received saline alone (0.4 mL, i.p.); (**D**) FIP exposed VitC deficient Gulo^−/−^ mice [**FIP**(−)] mice received fecal stem solution (45 mg/mL, i.p.) followed 30 min later by saline (0.1 mL, i.p.). (**E**) AscA treated FIP exposed VitC deficient Gulo^−/−^ mice [**FIP**(−) + **AscA**] mice received fecal stem solution (45 mg/mL, i.p.) followed 30 min later by AscA (200 mg/kg, i.p.). Platelet CD-41 (green), histones (red), and myeloperoxidase (grey) are seen in the merged images. Arrowheads indicate NET formation shown by co-staining for platelet CD-41 (green), histones (red), myeloperoxidase (grey), and DAPI (blue) in the vascular and alveolar spaces. Arrows indicate extensive extra-nuclear histones (red); (10× magnification, *N* = 3 for each group). (**F**) Serum levels of cf-DNA were quantified using the Quant-iTPicoGreen dsDNA assay kit (*N* = 5–11 for each group, *p* < 0.05).

## 3. Results

### 3.1. Vitamin C Sufficient Mice Demonstrate Reduced Lung NETs and Lower cf-DNA Following Peritonitis-Induced Sepsis

We have previously shown that fecal peritonitis promotes PMN infiltration of the lungs in VitC deficient mice [19]. Here we used immunofluorescence staining and DIC microscopy to examine the extent of NETs in lungs of mice following FIP induced sepsis. Immuno-positive staining for platelet CD-41 (green), nuclear histones (red), and myeloperoxidase (grey) are visible in the lungs of saline exposed mice (Figure 1A). No appreciable immuno-positive staining differences were seen in the lungs of saline exposed VitC deficient mice (Figure 1C). FIP induced a mild increase in CD-41 immuno-positivity as well as some cytosolic histone staining (Figure 1B). However, no significant histological changes were evident in the VitC sufficient septic mice. In contrast, FIP induced significant NETs in VitC deficient mice as evidenced by dramatically increased co-staining for platelet CD-41 (green), histones (red), and myeloperoxidase (grey) in the vascular and alveolar spaces of septic mice (arrowheads, Figure 1D). Moreover, extensive extra-nuclear staining of histones (arrows) is also evident in this representative section along with thickened alveolar walls. Importantly, FIP exposed vitamin C deficient mice treated with ascorbic acid exhibited significant attenuation of NETs (Figure 1E).

In order to quantify NETs we determined levels of cf-DNA in the serum of VitC sufficient and deficient mice 16 h after sham treatment or FIP. Levels of serum cf-DNA were significantly elevated in the FIP exposed VitC deficient mice (Figure 1F, 5-fold, *p* < 0.05). Treatment of septic VitC deficient mice with ascorbic acid significantly lowered the cf-DNA values to control levels (*p* < 0.05). In addition peritoneal neutrophils from vitamin C deficient mice were more susceptible to NETosis than those from vitamin C deficient mice (Supplementary Material, Figure S1).

### 3.2. Vitamin C Deficient Neutrophils Show Increased PAD4 mRNA

**Figure 2:**
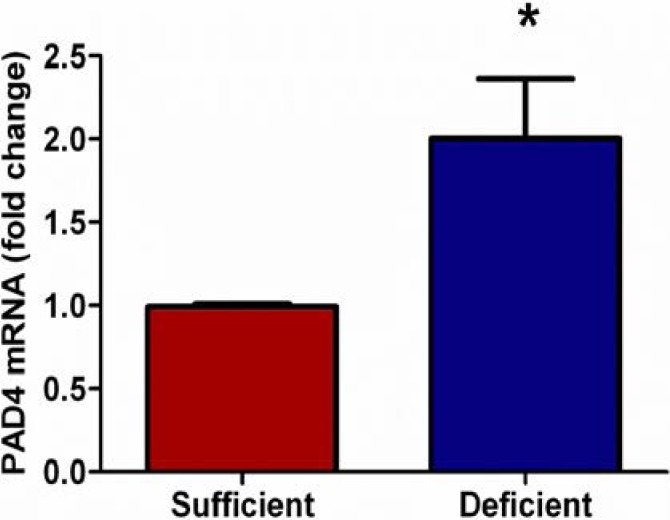
Vitamin C deficient neutrophils show increased PAD4 mRNA. Real time QPCR for PAD4 shows two-fold increase in mRNA expression from peritoneal PMNs of VitC deficient Gulo^−/−^ mice when compared to PMNs from VitC sufficient Gulo^−/−^ mice (*N* = 6 for each group, * *p* < 0.05).

Unlike apoptosis, rapid intracellular decondensation of nuclear chromatin is a hallmark of NETosis [19,25]. Decondensation of nuclear chromatin requires the removal of positively charged arginine residues on histones by deimination or citrullination, which is carried out by a family of peptidylargininedeiminases (PAD). Of these, only PAD4 is expressed by neutrophils [26] and possesses a classical nuclear localization signal [27]. Importantly Wang *et al.* have shown that PAD4 is indispensable for NETosis [18]. Therefore, we examined mRNA expression of PAD4 in PMNs from VitC sufficient and deficient mice. As seen in Figure 2, PAD4 mRNA expression was significantly higher in thioglycollate elicited peritoneal PMNs from VitC deficient mice (*p* < 0.05).

### 3.3. Autophagy Signaling Is Induced in Vitamin C Deficient Neutrophils

**Figure 3:**
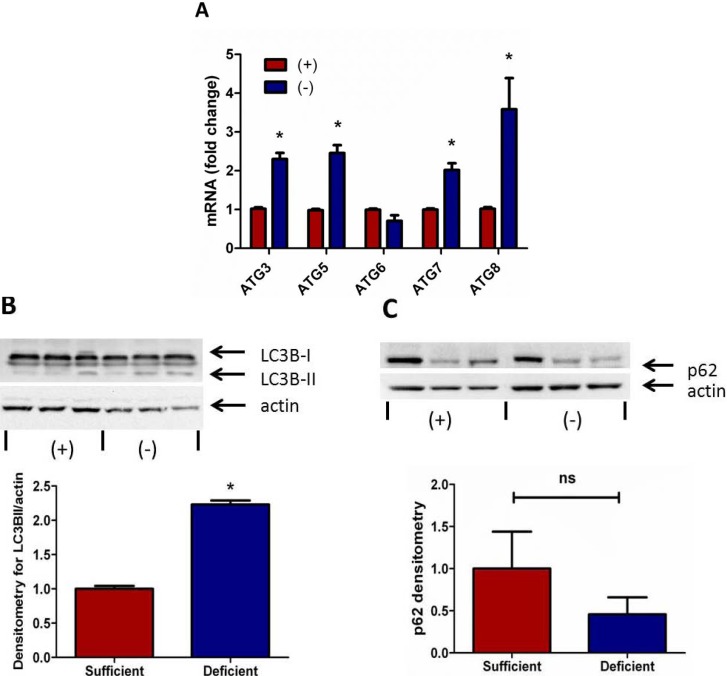
Autophagy signaling is induced in Vitamin C deficient neutrophils. (**A**) Real time QPCR for ATG3, ATG5, ATG6, ATG7, and ATG8 mRNA from peritoneal PMNs of VitC sufficient and deficient Gulo^−/−^ mice, (*N* = 6 for each group, * *p* < 0.05). (**B**) Representative Western blot for expression of LC3B-I and LC3B-II from peritoneal PMNs of VitC sufficient and deficient Gulo^−/−^ mice. Densitometry of LC3B-II/actin from peritoneal PMNs of VitC sufficient and deficient Gulo^−/−^ mice (*N* = 6 for each group, * *p* < 0.05). (**C**) Representative Western blot for expression of p62 and actin from peritoneal PMNs of VitC sufficient and deficient Gulo^−/−^ mice. Densitometry of normalized p62 expression from peritoneal PMNs of VitC sufficient and deficient Gulo^−/−^ mice (*N* = 6 for each group, ns *p* = 0.3).

Autophagy is a vital process for the catabolism of cytosolic proteins and organelles, but has also been shown to be required for NETosis [17,28]. To examine whether VitC regulates autophagy in PMNs we assessed the expression of several autophagy genes in thioglycollate elicited PMNs from VitC sufficient and deficient mice. As seen in Figure 3A, the expression of autophagy related signaling molecules (except for ATG6) were significantly elevated in the VitC *deficient* PMNs (*p* < 0.05).

Activation of the autophagic process causes lipidation of ATG8/LC3B (LC3B-I to LC3B-II conversion) and the lipid-modified LC3B-II translocates to autophagosomes. This LC3B-I to LC3B-II conversion is considered a critical marker of autophagy activation [29]. We observed significantly enhanced LC3B-I to LC3B-II conversion in cell lysates of VitC *deficient* PMNs by immunoblotting (Figure 3B, *p* < 0.05).

To further investigate the regulation of autophagy signaling by VitC in PMNs we examined the accumulation of p62/sequestosome I in these cell lysates. The loss of p62 in cells is typically indicative of increased autophagic activity [30]. Detection of p62 by immunoblotting showed a trend towards decreases p62 levels in the VitC *deficient* PMNs (Figure 3C). However this decline did not reach statistical significance (*p* = 0.3).

**Figure 4:**
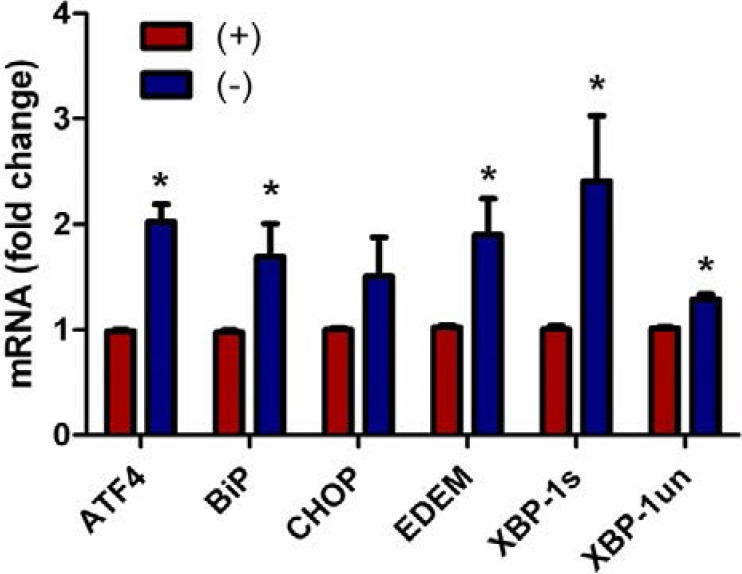
Endoplasmic reticulum stress associated gene expression in up-regulated in vitamin C deficient neutrophils. Real time QPCR for activating transcription factor 4 (ATF4), glucose-regulated protein 78 (Grp78, BiP), C/EBP homologous protein (CHOP), ER degradation-enhancing α-mannosidase-like protein (EDEM), X-box binding protein-1 spliced (XBP-1s), and unspliced (XBP-1un) mRNA from peritoneal PMNs of VitC sufficient and deficient Gulo^−/−^ mice, (*N* = 6 for each group, * *p* < 0.05).

### 3.4. Endoplasmic Reticulum Stress Associated Gene Expression Is Up-Regulated in Vitamin C Deficient Neutrophils

Signaling initiated by the ER stress response (unfolded protein response, UPR) actively participates in autophagy and ultimately contributes to the cell fate decision [31]. Since autophagy signaling was induced in the VitC deficient PMNs, we next examined ER stress gene expression in the PMNs. As seen in Figure 4, all the UPR genes examined except for CHOP were significantly up-regulated in PMNs from VitC *deficient* mice (*p* < 0.05).

### 3.5. Vitamin C Deficient Neutrophils Undergo Attenuated Apoptosis

**Figure 5:**
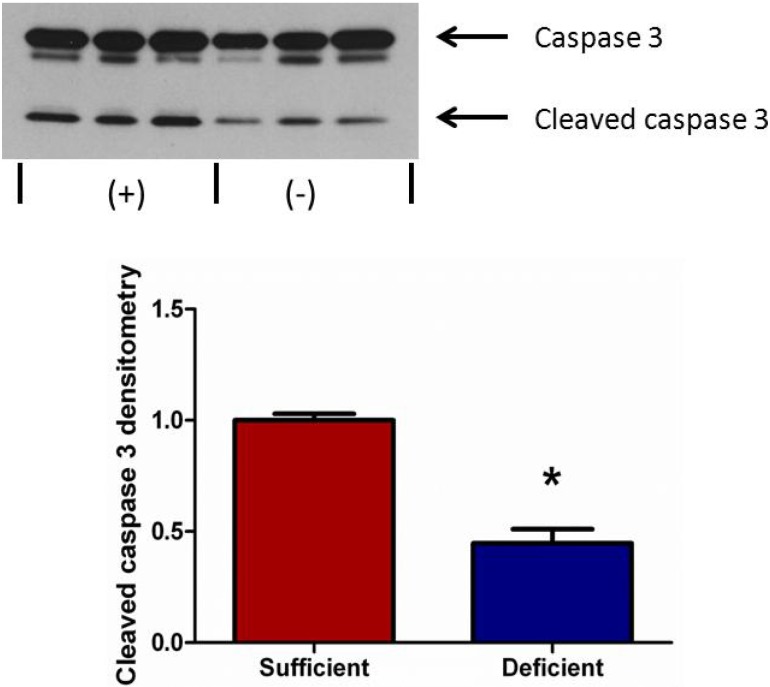
Vitamin C deficient neutrophils undergo attenuated apoptosis. Representative Western blot for expression of caspase-3 and cleaved caspase-3 from peritoneal PMNs of VitC sufficient and deficient Gulo^−/−^ mice. Densitometry of cleaved caspase-3/caspase-3 from peritoneal PMNs of VitC sufficient and deficient Gulo^−/−^ mice (*N* = 6 for each group, * *p* < 0.05).

To determine the extent of apoptosis in peritoneal PMNs from VitC sufficient and VitC deficient mice, we examined a well characterized marker of apoptosis, cleaved caspase-3, by immunoblotting of PMN cell lysates. As seen in Figure 5, caspase-3 activation was significantly lower in VitC *deficient* PMNs (*p* < 0.05).

### 3.6. Vitamin C Deficient Neutrophils Exhibit Increased NFκB Activation

**Figure 6:**
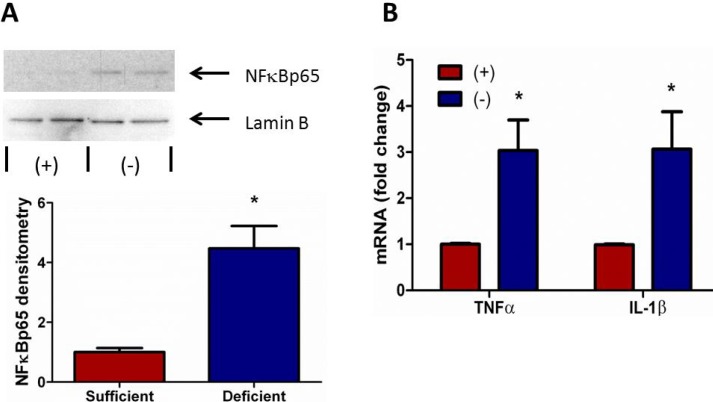
Vitamin C deficient neutrophils exhibit increased NFκB activation. (**A**) Representative Western blot for nuclear expression of NFκBp65 and Lamin B from peritoneal PMNs of VitC sufficient and deficient Gulo^−/−^ mice. Densitometry of NFκBp65/Lamin B from peritoneal PMNs of VitC sufficient and deficient Gulo^−/−^ mice (*N* = 4 for each group, * *p* < 0.05). (**B**) Real time QPCR for TNFα and IL-1β mRNA from peritoneal PMNs of VitC sufficient and deficient Gulo^−/−^ mice, (*N* = 6 for each group, * *p* < 0.05).

The transcription factor NFκB modulates the expression of many immuno-regulatory mediators in the acute inflammatory response in sepsis. Yang *et al.* found that diminished nuclear translocation of NFκB in peripheral PMNs was associated with less time on the ventilator and improved survival in critically ill patients [32]. NFκB activation is also associated with increased ROS production and endoplasmic reticulum stress signaling [33]. Therefore, we examined nuclear translocation of NFκB in peritoneal PMNs isolated from VitC sufficient and deficient mice. As seen in Figure 6A, significantly increased NFκB translocation was observed in nuclei of VitC *deficient* PMNs (*p* < 0.05). Increased nuclear NFκB translocation was associated with induction of the NFκB dependent pro-inflammatory genes for TNFα and IL-1β (Figure 6B, *p* < 0.05).

### 3.7. Vitamin C Attenuates NET Formation in Activated Human Neutrophils

**Figure 7:**
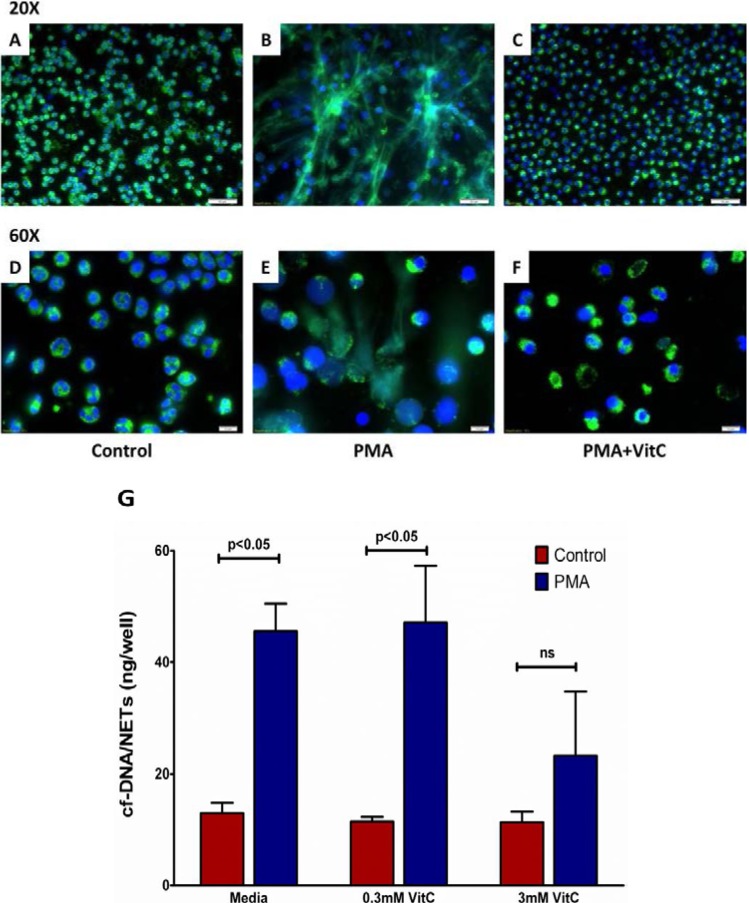
Vitamin C attenuates NET formation in activated human neutrophils. Representative image of immunofluorescent staining for NETs in human neutrophils: DNA (blue); myeloperoxidase (green). **Upper Panels**: Control PMNs (**A**, 20×); PMNs exposed to PMA (50 nM) for 3 h (**B**, 20×); PMNs loaded with VitC (3 mM) for 1 h and then exposed to PMA (50 nM) for 3 h (**C**, 20×). **Lower Panels**: Control PMNs (**D**, 60×); PMNs exposed to PMA (50 nM) for 3 h (**E**, 60×); PMNs loaded with VitC (3 mM) for 1 h and then exposed to PMA (50 nM) for 3 h (**F**, 60×). (*N* = 3 for each group, Magnification: upper panel 20×, lower panel 60×). (**G**) Quantification of cf-DNA in the supernatants above (*N* = 3 for each group, *p* < 0.05).

Freshly isolated human PMNs formed NETs following activation by PMA (50 nM) for three hours as seen by immunofluorescence staining (Figure 7B,E). Loading the cells with VitC (3 mM) prior to PMA stimulation greatly reduced NET formation by human PMN (Figure 7C,F). Further, quantification of cf-DNA from the supernatants showed VitC (3 mM) loading significantly reduced NETs release from activated PMN (Figure 7G, *p* < 0.05).

## 4. Discussion

In this study we show that VitC could play a critical role in regulating the ultimate fate of PMNs in sepsis. Activated PMNs undergo extensive NETosis in septic mice lungs, resulting in potential damage to lung alveolar and endothelial cells. This effect was predominant in PMNs from VitC deficient mice and could be rescued by VitC infusion after the onset of sepsis. In contradistinction, PMNs from VitC sufficient mice underwent attenuated NETosis. Importantly, at a molecular level, VitC deficient peritoneal PMNs were likely to be more pro-inflammatory, to resist apoptosis, and to preferentially undergo NETosis.

Although several signaling mechanisms responsible for NET formation have been reported, critical regulatory elements remain unidentified. This study advances our understanding of PMN function and NET biology by identifying a novel regulatory mechanism for NET formation in both murine and human PMNs. Using our previously well-characterized model of abdominal peritonitis induced sepsis we show that sepsis promotes NET formation in lungs of VitC deficient mice (Figure 1). NETosis in this model was accompanied by increased circulating cf-DNA (Figure 1F). VitC sufficiency or infusion of VitC after initiation of sepsis significantly decreased NETosis in lungs and circulating cf-DNA content (Figure 1). NET formation in VitC deficient peritoneal PMNs required activation of well characterized signaling pathways including ROS generation (data not shown), activation of the peptidylargininedeiminase PAD4 (Figure 2), autophagy (Figure 3), endoplasmic reticulum stress (Figure 4), and inhibition of apoptosis (Figure 5). NFκB, a pro-inflammatory, pro-survival transcription factor was activated in the VitC deficient peritoneal PMNs (Figure 6). VitC sufficiency or treatment with VitC attenuated these signaling pathways in PMNs.

Intracellular chromatin decondensation is essential for NET formation. Chromatin decondensation is brought about by peptidylargininedeiminase 4 (PAD4), a nuclear enzyme that deiminates arginine residues on histone tails thereby converting positively charged arginines to uncharged citrullines [17,18]. The importance of PAD4 is that many NET forming stimuli including PMA, LPS, and IL-8 as well as various bacterial and fungal species converge to its activation. While PAD4 is expressed in PMNs and is localized to the nucleus [26,27], little is known about its mechanism of action or its transcriptional regulation. Ying *et al.* have shown that PAD1, which belongs to the same family of enzymes as PAD4, is transcriptionally regulated by NFκB [34]. We have previously shown that VitC blocks NFκB activation in septic mouse lungs [12]. Cárcamo *et al*. also demonstrated that VitC blocks IκB kinase activity and NFκB activation [35]. In this study we observed that nuclear NFκB levels were higher in the VitC deficient PMNs (Figure 6A). Further, PAD4 mRNA expression was also significantly higher in PMNs from VitC deficient mice (Figure 2). Therefore, we hypothesize that VitC decreases PAD4 expression by suppressing NFκB activation in PMNs. Further, by decreasing PAD4 expression VitC could decrease histone citrullination activity and therefore chromatin decondensation in VitC sufficient PMNs.

Autophagy has been identified as a well-conserved, homeostatic mechanism that clears damaged organelles or proteins and plays an essential role in cell survival during periods of nutrient depletion [36]. Despite the view that it might not occur in neutrophils, autophagy was recently shown to occur both in murine and human PMNs [37,38]. While Mitroulis *et al*. reported that autophagy occurs in human PMNs in response to PMA activation [39]. Remijsen *et al*. have shown that autophagy is necessary for the induction of intracellular chromatin decondensation during PMA-induced NETosis [17]. In our study, we found increased expression of autophagy genes (Figure 3A) as well as significantly enhanced LC3B-I to LC3B-II conversion in VitC deficient PMNs (Figure 3B) indicative of the presence of more autophagosomes in VitC deficient PMNs. However, LC3B-I to LC3B-II conversion is a static measure of autophagosome number, and does not measure the actual activity of the pathway. The increased LC3B-II could be interpreted as either high autophagic activity or a downstream block in the system that results in an accumulation of LC3B-II protein, even though autophagic degradation itself is diminished. To supplement our observations we examined levels of p62/sequestosome I, a cytosolic chaperone protein with an LC3B binding domain [40]. The normal function of p62 protein is to carry polyubiquitinated proteins to the autphagolysosome where it binds to LC3B before getting degraded. Thus, the loss of p62 protein is a measure of the flux of autophagy and indicative of increased autophagy [41]. In our studies we found a trend towards decreased p62 levels in the VitC deficient PMNs (Figure 3C). While this decline did not reach statistical significance, in combination with the increased autophagy gene expression and increased LC3B conversion, our data imply increased autophagy in VitC deficient PMNs.

The unfolded protein response (UPR) and autophagic machinery have been shown to be critically linked to each other. It is well established that activation of the UPR genes transcriptionally up-regulates several autophagy related genes required for induction and construction of the autophagy machinery [42]. However, it is not known whether activation of the UPR drives autophagy and eventually leads to NET formation in PMNs. Our study shows that most of the UPR genes examined except for CHOP were significantly up-regulated in PMNs from VitC deficient mice (Figure 4). This implies that VitC deficient PMNs could be actively undergoing ER stress, which in turn could drive autophagy genes and increase their susceptibility to undergo NETosis.

**Figure 8:**
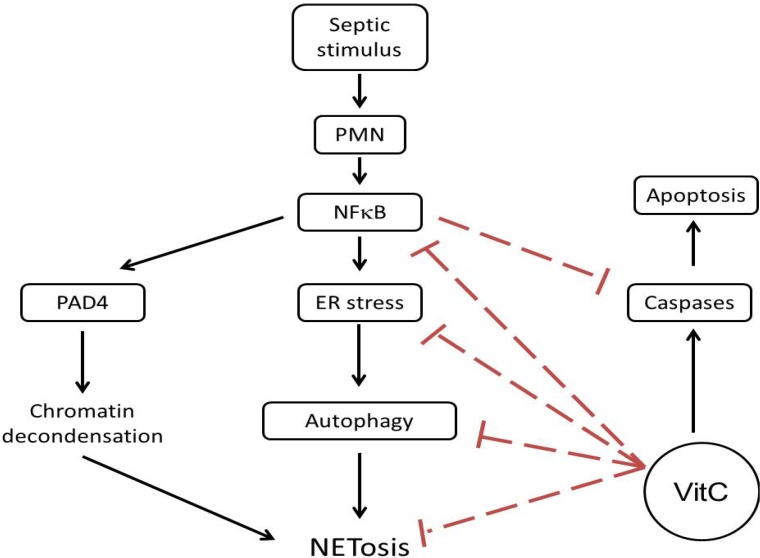
Schematic hypothesis of regulation of signaling pathways that leads to NETosis by VitC. Septic stimuli activate NFκB in PMNs with increased activation observed in VitC deficient PMNs. NFκB nuclear translocation drives expression of PAD4, ER stress and autophagy signaling genes while inhibiting caspase 3 in activated PMNs. This drives the fate of activated PMN away from apoptosis and enhances NETosis. VitC likely blocks up-regulation of PAD4, ER stress and autophagy signaling genes by decreasing NFκB activation. Activated PMNs now undergo apoptosis while NETosis is attenuated.

The transcription factor NFκB is central to pro-inflammatory/pro-survival responses in sepsis. It is normally sequestered to IκB in the cytosol. Upon appropriate stimulation, IκB is degraded allowing NFκB to migrate to the nucleus and drives transcription of numerous genes that regulate the immune response in sepsis. Moine *et al*. have demonstrated increased NFκB translocation in the lungs of patients with ALI [43]. Yang *et al*. found that increased nuclear levels of NFκB in unstimulated neutrophils were associated with a worse clinical outcome [32]. As discussed above, NFκB likely drives expression of PAD4 in PMNs. NFκB activation also drives expression of pro-survival genes [44]. In this study we found that nuclear NFκB translocation was higher in VitC deficient PMNs (Figure 6A). Further, NFκB translocation in these VitC deficient PMNs increased expression of the pro-inflammatory genes TNFα and IL-1β (Figure 6). NFκB activation also inhibited apoptosis as seen by the reduced activation of caspase 3 in VitC deficient PMNs (Figure 5). These results suggest that NFκB may play a critical role in modulating cell signaling pathways that eventually regulate the fate of PMNs. By activating PAD4 (chromatin decondensation), inducing ER stress and subsequent autophagy, and inhibiting apoptosis, NFκB may drive the cellular machinery of VitC deficient PMNs towards NET formation (Figure 8). VitC sufficiency or infusion of VitC allows PMNs to increase intracellular levels of VitC and attenuate NFκB activation. This could dampen the pathways required for NETosis and may allow PMNs to undergo apoptosis instead. While the decreased apoptosis rate in VitC deficient PMNs may benefit the host by giving more time for PMNs to perform their innate immune functions, studies show that it could also be detrimental in sepsis due to the PMN-dependent inflammation and tissue damage that could be heightened by a prolonged lifespan. Recent reports in the literature have implicated NETs in transfusion-related acute lung injury (TRALI), the leading cause of death after transfusion therapy [45,46]. NETs were shown to be present during TRALI both in mice and humans and so it was suggested that targeting NET formation may be a new approach for the treatment of acute lung injury. While we did not examine TRALI in our studies, it is conceivable that VitC infusion could be a useful adjunct for the prevention/treatment of TRALI or other disease states involving exuberant formation of NETs particularly in the lungs.

Our study has several limitations: (1) It is possible that the PMNs isolated within the peritoneal cavity by thioglycollate could be partially activated; (2) We examined PMN function *ex vivo*. Further *in vivo* studies are needed to characterize the fate of PMNs; (3) others have performed studies with PMNs isolated from bone marrow instead of thioglycollate elicitation. These PMNs would be less “activated” when compared to thioglycollate elicited PMNs, but would also have a large component of immature PMNs which have been shown to behave somewhat differently from mature PMNs [47].

## 5. Conclusions

In the past few years circulating cf-DNA has been identified as a prognostic marker in severe sepsis [11,48,49]. Indeed cf-DNA was shown to have better discriminatory power than IL-6, thrombin or protein C to predict ICU mortality in sepsis [11]. The cellular origin of cf-DNA from host cells was shown by Dwivedi *et al.* [11] who confirmed that the release of cf-DNA was independent of the infecting organism and was likely mediated by inflammatory mediators generated during the exacerbated host immune response. Our study showed attenuated NET formation and reduced cf-DNA in the serum of septic VitC sufficient mice and in VitC deficient mice treated with ascorbic acid (Figure 1). Our study did not examine the origin of cf-DNA in the serum of these mice. It is possible that some of this DNA could be non-neutrophilic in origin since mast cells, eosinophils, and basophils have also been shown to expel their DNA in a manner similar to PMNs [50]. However, a detailed examination of the origin of cf-DNA in these septic mice is beyond the scope of this study. Nevertheless, data from our study implies that attenuation of NETs maybe crucial for resolution of sepsis in mice.

Overall, our *in vitro* and *in vivo* findings identify a novel regulatory mechanism that limits NET formation in sepsis. These findings implicate VitC as a previously unrecognized layer of regulation that prevents generation of excessive NETs.

## Supplementary Files

Supplementary File 1 Supplementary Information (DOCX, 71 KB)
