# Supplementary material for: Vitamin C: A Novel Regulator of Neutrophil Extracellular Trap Formation
Source: Nutrients. 2013 Aug 9;5(8):3131–50. doi: 10.3390/nu5083131 (PMC3775246; doi:10.3390/nu5083131)

**Supplementary Information**

Vitamin C (VitC) deficient neutrophils are susceptible to NETosis. In order to determine whether peritoneal neutrophils from VitC sufficient or VitC deficient mice produced NETs without further stimulation, thioglycollate-elicited peritoneal PMNs were seeded onto 8-well IbiTreat µ-slides (Ibidi #80826) and allowed to adhere for 16 h. As described in the Methods section of the manuscript, PMNs were fixed with 4% paraformaldehyde, permeabilized with 0.15% Triton X-100 in PBS, and blocked with 5% normal chicken serum (Sigma) in PBS. To stain NETs, slides were incubated with a mouse monoclonal anti-myeloperoxidase (MPO) antibody and a secondary Alexa Fluor^®^ 488-conjugated chicken anti-mouse IgG antibody. After staining of DNA with DAPI, PMNs were visualized by immunofluorescence microscopy. As seen below (Figure S1a), PMNs from VitC sufficient mice retained their lobulated structure with no significant co-localization of DNA and MPO signals (no inter-mixing of nuclear and cytosolic contents). In contrast, PMNs from VitC deficient mice demonstrated some degree of co-localization of DNA and MPO signals (Figure S1b). Importantly, nuclei from some of these PMNs (arrows) were delobulated and had released their DNA content into the cytosol. However, full NET structures are not evident in these images. This suggests that thioglycollate stimulation of PMNs is insufficient to induce NETs in murine PMNs from VitC sufficient mice. Conversely, some PMNs from VitC deficient mice were sufficiently activated and had commenced the process of NET formation by intermixing nuclear and cytosolic contents.

**Figure S1.** Vitamin C deficient neutrophils are susceptible to NETosis. Representative image of immunofluorescent staining of Thioglycollate elicited peritoneal PMNs (DNA (blue); myeloperoxidase (green)) obtained from: (**a**) VitC sufficient mice; nuclei from VitC sufficient mice retained their lobulated structure, and (**b**) VitC deficient mice; PMNs from VitC deficient mice demonstrated some degree of co-localization of DNA and MPO signals. Nuclei from some of these PMNs (arrows) were delobulated and had released their DNA content into the cytosol.


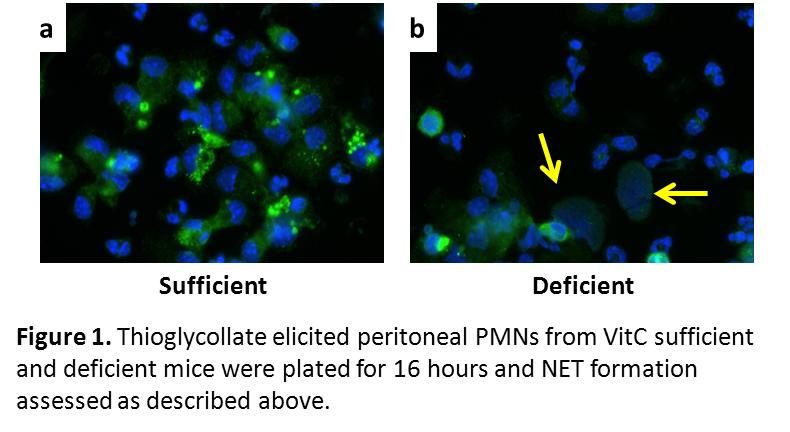

Supplement: Supplementary File 1 — Supplementary Information (DOCX, 71 KB) [file nutrients-05-03131-s001.docx]
